# Fordyce angiokeratoma

**DOI:** 10.11604/pamj.2014.19.376.5874

**Published:** 2014-12-12

**Authors:** Theocharis Koufakis, Ioannis Gabranis

**Affiliations:** 1 Department of Internal Medicine, General Hospital of Larissa, Larissa, Greece

**Keywords:** Fordyce angiokeratoma, scrotum, papules

## Image in medicine

A 55 year-old Caucasian man, with Diabetes Melitus type 2 history, presented to our department with complaints of multiple, dark red papules located on the scrotum. The diagnosis of Fordyce angiokeratoma was clinically established. No special treatment was recommended, apart from patient's reassurance for the benign nature of this clinical entity. Fordyce angiokeratoma or angiokeratoma of the scrotum was first described in 1896 by John Addison Fordyce. Its etiology remains unclear, while its incidence is believed to increase with age. Genetic background of the disease has not been documented. Complications are generally uncommon, however bleeding of the papules can occur. Differential diagnosis mainly includes angiokeratoma corporis diffusum, malignant melanoma and nevomelanocytic nevus. In cases of diagnostic dilemma, dermoscopy can be useful, but biopsy of the lesion is the gold standard method for the establishment of the diagnosis. Specific treatment is unnecessary, still, excision, electrodessication and laser therapy have been described in complicated cases or for aesthetic reasons.

**Figure 1:**
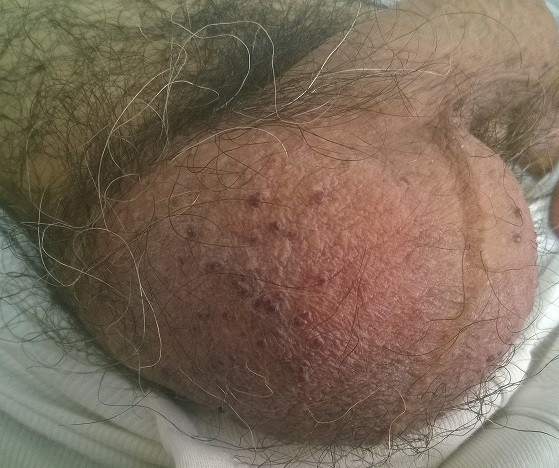
Fordyce Angiokeratomas on the scrotum

